# Ischemic Colitis Secondary to Mesenteric Arteriovenous Malformation: A Rare Cause of Bowel Ischemia—Case Report and Literature Review

**DOI:** 10.1002/deo2.70289

**Published:** 2026-02-24

**Authors:** Arparat Kanacharoen, Nantaporn Srivanitchapoom, Pornrujee Hirunpat, Nuttapat Tungtrongchitr

**Affiliations:** ^1^ Chakri Naruebodindra Medical Institute, Faculty of Medicine, Ramathibodi Hospital Mahidol University Samut Prakan Thailand

**Keywords:** arteriovenous malformation, ischemic colitis, lower gastrointestinal bleeding, mesenteric arteriovenous malformation, mesenteric ischemia

## Abstract

Ischemic colitis is the most common form of intestinal ischemia, typically caused by a temporary reduction in blood flow to the colon. It is often linked to atherosclerosis, low blood pressure, or embolism. We present a case of ischemic colitis with atypical location due to an inferior mesenteric arteriovenous malformation (inferior mesenteric artery–vein) in a 77‐year‐old woman with Child‐Turcotte‐Pugh Class A hepatitis C cirrhosis. She had a 2‐day history of rectal bleeding, mild abdominal pain, and nausea. Colonoscopy findings show segmental erythematous and edematous mucosa with multiple discrete shallow ulcers in the sigmoid and descending colons (23–30 cm from the anal verge). Computed tomography (CT) and CT angiography showed an arteriovenous malformation (AVM) supplied by the inferior mesenteric artery, draining into the inferior mesenteric vein, consistent with ischemic colitis caused by a steal phenomenon. Given the high risk of worsening ischemia with embolization, the patient underwent surgical resection and ligation of the AVM, with complete resolution of symptoms postoperatively. This case highlights the importance of considering mesenteric AVMs in the diagnosis of ischemic colitis, particularly when classic risk factors are absent, and underscores the role of early CT angiography in facilitating prompt diagnosis and treatment.

## 1 Introduction

Ischemic colitis is the most commonly found form of gastrointestinal ischemia, accounting for 50%–60% of gastrointestinal ischemic events and comprising up to 24% of hospitalized cases of acute lower gastrointestinal bleeding [1, 2]. Its incidence ranges from 4.5 to 44 per 100,000 person‐years. It increases with age, particularly among women over 65 [2, 3]. Ischemic colitis results from transient hypoperfusion, most often in the setting of systemic hypotension, atherosclerosis, or thromboembolic disease [4]. Endoscopically, the disease appears as segmental erythema, oedema, friability, and shallow colon ulcerations, typically confined to the “watershed” areas—the splenic flexure and rectosigmoid junction [1, 5].

Most cases of ischemic colitis are non‐occlusive and resolve with supportive care, but rarely, vascular anomalies like mesenteric arteriovenous malformations (AVMs) can cause mucosal hypoxia through a steal phenomenon. AVMs of the inferior mesenteric artery–vein (IMA–IMV) axis are especially rare. Diagnosis requires a high index of suspicion and is best confirmed with computed tomography (CT) angiography. Here we present a case of ischemic colitis secondary to a mesenteric AVM.

## 2 Case Report

A 77‐year‐old woman with hepatitis C‐related cirrhosis (Child–Turcotte–Pugh A) presented with a two‐day history of hematochezia and mild left‐sided abdominal pain. She did not have a fever or vomiting, and she had no history of gastrointestinal bleeding before this episode. Her surgical history included a total abdominal hysterectomy for a uterine myoma 40 years earlier. She was taking carvedilol (6.25 mg twice daily) and had no family history of malignancy.

On admission, the patient's vital signs were stable and afebrile. Physical examination showed conjunctival pallor and mild left‐sided abdominal tenderness without peritonitis. No stigmata of portal hypertension or ascites were noted. Laboratory testing (Table 1) revealed leukocytosis, mild normocytic anemia, and a slightly prolonged prothrombin time, with normal liver and renal functions. Stool cultures and Clostridioides difficile toxin assays were negative.

**TABLE 1:** Laboratory results of the patient.

Lab test	Reference range	Unit	Result
Blood urea nitrogen	10–20	mg/dL	18
Creatinine	0.55–1.02	mL/min/1.73 m^2^	0.95
Electrolytes			
Sodium	136–145	mmol/L	140
Potassium	3.5–5.1	mmol/L	3.5
Chloride	98–107	mmol/L	108
Carbon dioxide	22.0–29.0	mmol/L	22
Fasting blood glucose	74–100	mg/dL	110
HBA1C	<6.5	%	5.54
Lipid profile			
HDL	>=60	mg/dL	46
LDL	<130	mg/dL	123
Triglyceride	<150	mg/dL	68
Cholesterol	<200	mg/dL	178
Liver function test			
AST	5–34	U/L	34
ALT	0–55	U/L	24
ALP	40–150	U/L	36
GGT	9–36	U/L	19
Albumin	34–48	g/L	36.5
Total protein	64–83	g/L	75
Total bilirubin	0.2–1.2	mg/dL	1.1
Direct bilirubin	0.0–0.5	mg/dL	0.4
Coagulogram			
APTT	23.4–30.6	sec	25.1
PT	9.9–11.9	sec	12.5
INR	0.9–1.1		1.15
TT	16–18.8	sec	16.6
C‐reactive protein	< 5	mg/L	63.94
Lactate	0.7‐2.5	mmol/L	0.9
Complete blood count			
WBC	4.00–10.00	×10^3^/µL	11.57 ↑
RBC	4.00–5.50	×10^6^/µL	4.00
HGB	12.00–16.00	g/dL	11.30 ↓
HCT	36.00–48.00	%	35.50 ↓
MCV	80.00–99.00	fL	88.6
MCH	27.0–31.0	pg	28.1
MCHC	33.0–37.0	g/dL	31.8 ↓
RDW	11.5–14.5	%	13.2
PLT	140.0–450.0	×10^3^/µL	130.0 ↓
MPV	7.20–11.10	fL	9.70
Neutrophils	40–74	%	73
Lymphocytes	19–48	%	19
Monocytes	3–9	%	7
Eosinophils	0–7	%	1
Basophils	0–2	%	0
RBC Morphology	Normal RBC		Normal
*C. difficile toxin A+B and GDH*	MRR		All Negative
CMV viral load	Negative	IU/mL	<34.5 (Negative)
ANTI‐HIV	Negative		Negative

**FIGURE 1:**
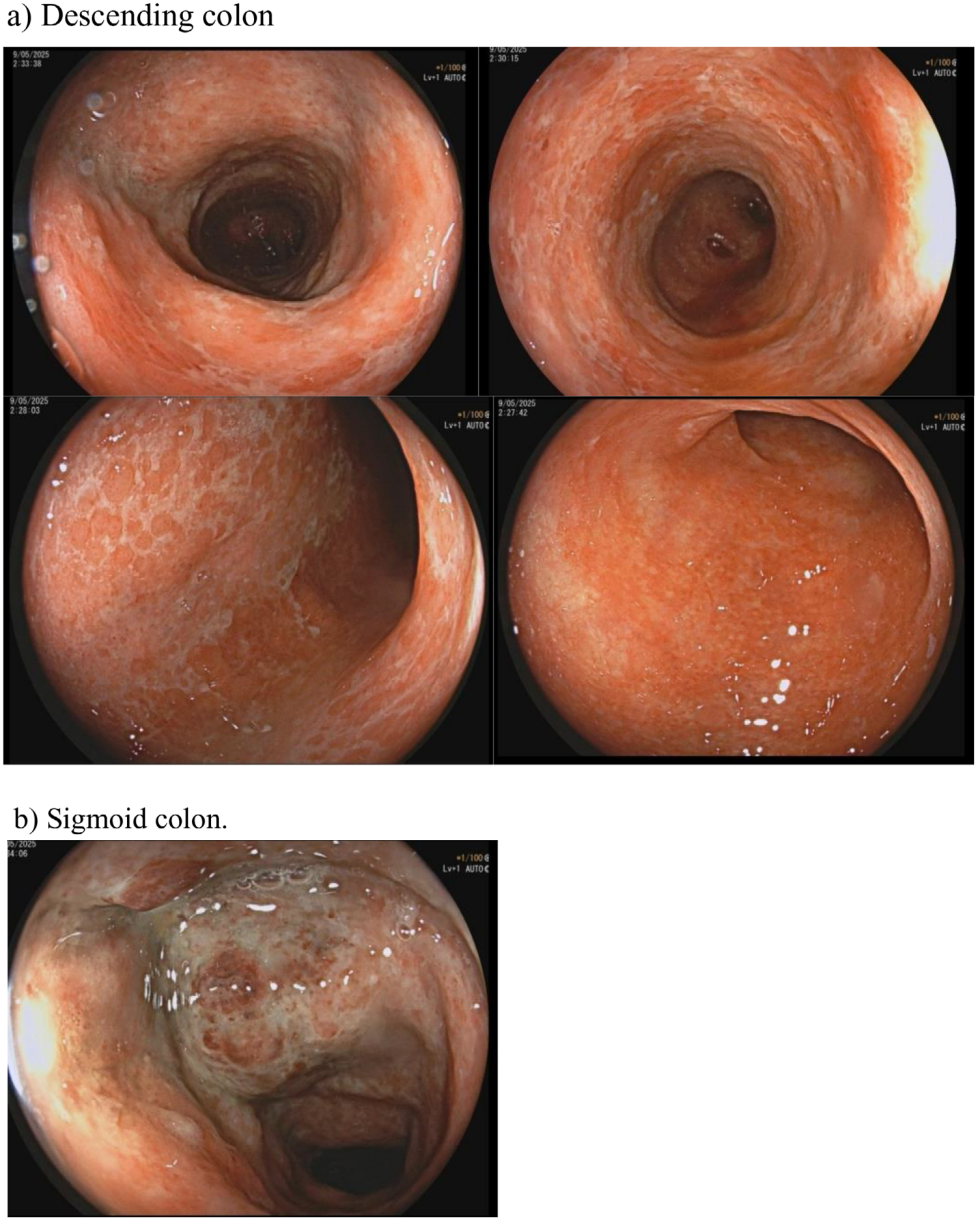
Findings from the colonoscopy in this case.

**FIGURE 2:**
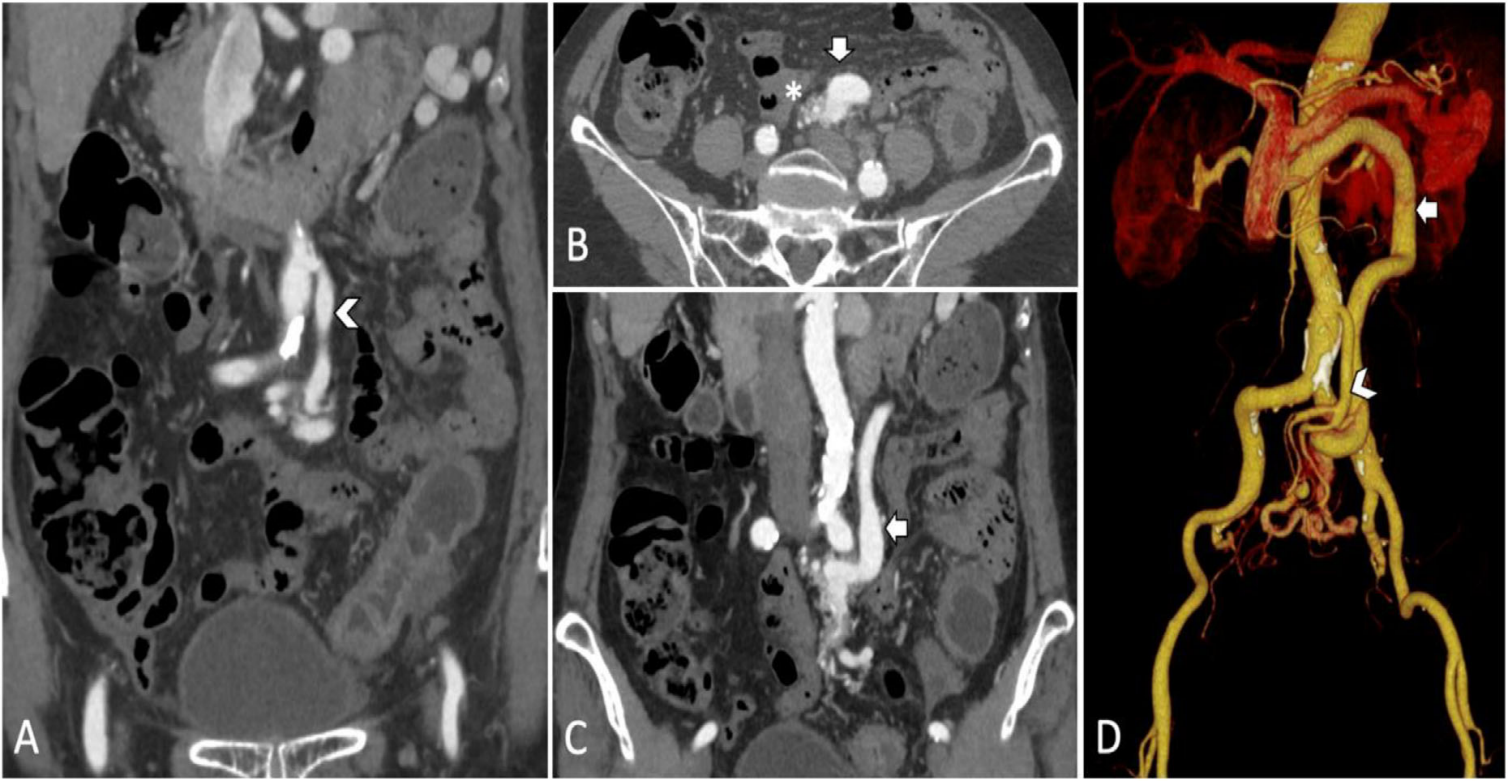
Findings of arteriovenous malformation from the computerized tomography of the abdomen and the computerized tomography angiogram in this case.

**FIGURE 3:**
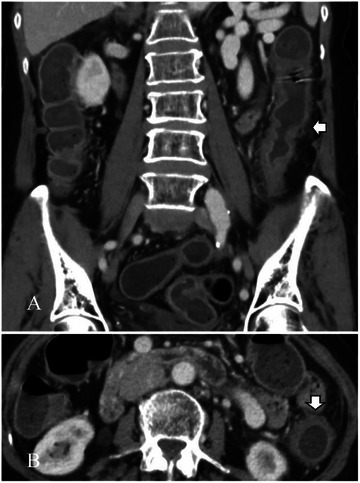
Findings of segmental colitis from the computerized tomography of the abdomen in this case.

A colonoscopy (Figure 1) performed one day post‐admission demonstrated circumferential erythema, oedema, friability, and shallow ulcerations in the descending and sigmoid colon (23–30 cm from the anal verge)—notably, the colitis extended beyond the classical watershed regions. Biopsies during colonoscopy revealed epithelial erosion and chronic active inflammation with reactive atypia. A contrast‐enhanced abdominal CT (Figures 2 and 3) done three days post‐admission revealed thickening and hypoenhancement of the descending and sigmoid colon. CT angiography identified an AVM originating from the IMA and draining into the IMV via a dilated outflow vein with a prominent vascular nidus, which was consistent with mesenteric steal physiology. This led to the diagnosis of ischemic colitis caused by a mesenteric AVM.

The patient was initially managed with absolute bowel rest, intravenous (IV) fluids, and empiric ceftriaxone 2 g IV once daily plus metronidazole 500 mg IV every 8 h. After a multidisciplinary discussion (involving gastroenterology, vascular surgery, and interventional radiology specialists), surgical resection was selected as the treatment modality over AVM embolization due to the high risk of worsening the colonic ischemia.

An open anterior resection with AVM ligation was performed nine days post‐admission. Intraoperative colonoscopy confirmed grade 1 ischemic colitis without transmural necrosis. The operative findings included a 4.0 cm mesenteric AVM with a 0.6 cm IMA feeding vessel and a 0.8 cm IMV draining vessel. Histopathology confirmed the presence of a mesenteric AVM and associated colonic mucosal epithelial erosion and chronic active inflammation. The patient's diet was initiated at two days post‐operatively, and recovery was uneventful. There was complete resolution of abdominal pain, and no further events of gastrointestinal bleeding, and the patient was discharged one week after the successful surgery. At a 3‐month follow‐up, the patient was well and had resumed a regular oral diet without any recurrent episodes of gastrointestinal bleeding or abdominal pain.

## 3 Discussion

The most common causes of ischemic colitis are atherosclerosis, thromboembolism, and hypotension [1, 2]; however, the present case differed from classic cases of ischemic colitis in several ways. The patient had no significant cardiovascular comorbidities or systemic hypotension at presentation, and colonoscopy showed diffuse involvement of the descending and sigmoid colon extending beyond the typical watershed zones. In usual cases of ischemic colitis due to atherosclerotic causes, colonoscopy findings often demonstrate segmental erythema, oedema, friability, and shallow ulcerations, most often confined to watershed zones such as the splenic flexure (Griffith's point, junction of the SMA and IMA) and rectosigmoid junction (Sudeck's point, junction of the IMA and hypogastric circulation) as these are the regions with marginal perfusion [6]. However, the colonoscopy findings in this case showed circumferential erythema, oedema, friability, and shallow ulcerations in the descending and sigmoid colon (23–30 cm from the anal verge), which did not follow typical watershed zones of poor perfusion, and thus, further CT angiography was prompted to assess for abnormal lesions in the arterial supply of the left‐sided colon.

In atypical cases of ischemic colitis, it's essential to differentiate the cause from other colonic inflammations, such as infectious colitis or inflammatory bowel disease. In this case, negative stool cultures and signs of ischemic injury ruled out these causes. A CT angiography revealed an AVM involving the inferior mesenteric artery and vein, which corresponded to the ischemic colitis observed in colonoscopy and imaging. This diagnosis of AVM‐induced ischemic colitis allowed for early surgical intervention before transmural necrosis could occur.

Mesenteric AVMs are rare vascular anomalies that can cause ischemia by diverting arterial blood from mucosal capillaries. They may present with bloody diarrhea and abdominal pain, resembling ischemic colitis, but colonoscopy findings alone may not identify the underlying cause.

CT angiography is crucial for diagnosing AVM‐related ischemic colitis, as it helps visualize the AVM nidus and feeding vessels. Without early CT angiography, AVM‐related ischemic colitis might be misclassified as idiopathic or atherosclerotic, delaying treatment and increasing the risk of complications like bowel perforation and sepsis.

Managing AVM‐related ischemic colitis is case‐specific and depends on the disease severity and anatomy. Despite a high recurrence rate, conservative medical treatment can be applied in mild or pauci‐symptomatic cases as demonstrated in Lakin's case report [7]. Endovascular embolization is becoming increasingly popular as a first‐line treatment, as it is less invasive than surgery [8]. Embolization may worsen ischemia if collateral circulation is lacking. In large fistulas over 8 mm, embolization material can migrate into the portal venous system [9]. In contrast, surgical resection is the preferred treatment for large AVMs or severe ischemia, yielding better long‐term outcomes and lower recurrence rates. A review of the literature on IMA‐IMV AVF case reports [10] found that out of 45% of patients who received initial surgical management, 35% received successful embolization, 7.5% were managed conservatively, while 12.5% required combined embolization and surgery following failure of single‐modality therapy.

Surgical treatment was chosen in this case for several reasons. The AVM measured 4.0 cm, which indicated a high blood flow. This made embolization riskier due to the potential for non‐target embolization and the migration of embolic materials, particularly in fistulas larger than 8 mm in diameter. Furthermore, conservative management carried a significant risk of recurrent bleeding and ischemic episodes. These complications could lead to necrosis, perforation, and sepsis if treatment is delayed. Therefore, surgical resection combined with AVM ligation was selected to eliminate the shunt, remove non‐viable bowel tissue, and reduce the risk of recurrence and disease progression.

This case illustrates a rare presentation of ischemic colitis secondary to an inferior mesenteric AVM in a patient without conventional risk factors. Unlike typical ischemic colitis, which is often located within watershed zones, this case involved diffuse left‐sided disease caused by mesenteric steal physiology. Performing a colonoscopy and CT angiography at an early stage enabled prompt diagnosis and treatment before complications of ischemia occurred. Clinicians should maintain a high index of suspicion for AVMs in cases of atypical ischemic colitis to ensure timely, definitive treatment.

## Author Contributions

**Arparat Kanacharoen**: conceptualization, data curation, formal analysis, and writing – original draft; **Nantaporn Srivanitchapoom**: visualization and writing – review & editing; **Pornrujee Hirunpat**: investigation, resources, and writing – review & editing; **Nuttapat Tungtrongchitr**: conceptualization, methodology, software, data curation, investigation, validation, formal analysis, supervision, project administration, and writing – review & editing

## Conflicts of Interest

The authors declare no conflicts of interest.

## Funding

None

## Ethics Statement

The patient consented. The Human Research Ethics Committee, Faculty of Medicine, Ramathibodi Hospital, Mahidol University, Bangkok, Thailand, reviewed and approved this study (COA. MURA 2025/550).
